# Nonfatal Occupational Injuries to Younger Workers — United States, 2012–2018

**DOI:** 10.15585/mmwr.mm6935a3

**Published:** 2020-09-04

**Authors:** Rebecca J. Guerin, Audrey A. Reichard, Susan Derk, Kitty J. Hendricks, Lauren M. Menger-Ogle, Andrea H. Okun

**Affiliations:** ^1^Division of Science Integration, National Institute for Occupational Safety and Health, CDC; ^2^Division of Safety Research, National Institute for Occupational Safety and Health, CDC.

Adolescents and young adults represent approximately 13% of the U.S. workforce (*1*). Compared with adult workers, young workers (aged 15–24 years) experience higher rates of job-related injury (*2*,*3*). To describe injuries among young workers and inform research and prevention activities, CDC’s National Institute for Occupational Safety and Health (NIOSH) analyzed national data for 2012–2018 from the occupational supplement to the National Electronic Injury Surveillance System* (NEISS-Work) and for 2018 from the Bureau of Labor Statistics (BLS) Survey of Occupational Injuries and Illnesses (SOII).^†^ During the 7-year period, an estimated 3.2 million (95% confidence interval [CI] = 2.6–3.7) nonfatal, job-related injuries to young workers were treated in hospital emergency departments (EDs). From 2012 to 2018, annual rates of work-related injuries^§^ treated in the ED (ED-treated injuries) declined overall across all age groups but ranged from 1.2 to 2.3 times higher for workers aged 15–24 years compared with those for adults aged 25–44 years. Workers aged 18–19 years had the highest rate of ED-treated injuries. In 2018, among all age groups, workers in service occupations^¶^ had the highest percentage of injuries requiring at least 1 day away from work. Among workers aged 15–17 years, those in the leisure and hospitality industry had the highest percentage of work-related injuries requiring at least 1 day away from work. Occupational injuries can have long-term impacts on health (*4*). The disproportionate risk of injury among young workers highlights the need for sustained, targeted public health efforts to prepare this population with essential workplace safety and health competencies before they enter the workforce and to provide high-quality safety training and close supervision on the job. NIOSH and its partners developed a free curriculum to teach adolescents workplace safety and health competencies, which includes identification of workplace hazards and methods for addressing them, how to understand their rights and responsibilities as workers, and how to voice concerns about work safety issues (*5*).

Data from NEISS–Work,** and the BLS SOII,^††^ the two main sources of national data on worker injuries,^§§^ were used for these analyses. NEISS-Work and SOII have substantially different methodologies for determining injury estimates (*2*) and together provide a more detailed picture of injuries to young workers. NEISS-Work data capture occupational injuries from a nationally stratified, statistically weighted probability sample of hospital EDs; however, standardized industry and occupation codes are not available for these data.^¶¶^

SOII captures federal and state injury and illness data from employers’ Occupational Safety and Health Administration logs,*** classified by industry^†††^ and occupation.^§§§^ SOII estimates are based on a statistically weighted probability sample of employer reports collected annually from approximately 230,000 private industry and public sector establishments.^¶¶¶^ The analysis of SOII data is limited to injury cases that required at least 1 day away from work. For both NEISS-Work and SOII, injury events or exposures are classified according to the Occupational Injury and Illness Classification System.****

NEISS-Work and SOII estimates for work-related injuries to workers aged 15–17 years (protected under child labor laws^††††^), 18–19 years, and 20–24 years were compared with estimates for workers aged 25–44 years.^§§§§^ NEISS-Work data were analyzed for the years 2012–2018 and the U.S. Census Bureau’s Current Population Survey^¶¶¶¶^ labor force denominator estimates were used to calculate annual rates (*1*). Average 7-year rates were calculated by dividing the sum of the yearly numerator estimates by the sum of the yearly denominator estimates. Variances of the estimates were pooled to calculate 95% CIs.***** BLS source data in SOII are not formulated for the customized age groups used in this analysis to allow for rate calculations and aggregate counts across years; therefore, only the most current year of data (2018) were included in the analysis. For SOII, relative standard errors were converted to 95% CIs.^†††††^ Because of missing race/ethnicity data (approximately 32% in NEISS-Work and 45% in SOII), injuries by race/ethnicity were not examined. Analyses were conducted using SAS statistical software (version 9.4; SAS Institute).

During 2012–2018, an estimated 12 million (95% CI = 9.7–14.2) occupational injuries to workers aged 15–44 years were treated in EDs with an average annual rate of 215 injuries per 10,000 full-time equivalent (FTE) workers (95% CI = 177–254). During the 7-year period, an estimated 3.2 million (95% CI = 2.6–3.7) nonfatal, job-related injuries to workers aged 15–24 years were treated in hospital emergency departments (Table 1). The highest injury rate (404 per 10,000 FTE) occurred among workers aged 18–19 years. Within each of the four age categories, the rate of injury was 1.4 to 1.5 times higher among males than among females (Table 1). Annual rates of injuries among young workers aged 15–24 years were 1.2–2.3 times higher than those for workers aged 25–44 years (Figure).

**TABLE 1:** National estimates and rates* for nonfatal occupational injuries treated in U.S. hospital emergency departments, by selected patient characteristics — National Electronic Injury Surveillance System occupational supplement, United States, 2012–2018

Characteristic	Age group of worker, yrs							
	15–17		18–19		20–24		25–44^†^	
	NE x1,000 (95% CI)	Rate per 10,000 (95% CI)	NE x1,000 (95% CI)	Rate per 10,000 (95% CI)	NE x1,000 (95% CI)	Rate per 10,000 (95% CI)	NE x1,000 (95% CI)	Rate per 10,000 (95% CI)
**Total**	**164 (131–197)**	**281 (223–339)**	**600 (484–716)**	**404 (325–482)**	**2,409 (1,980–2,838)**	**287 (236–337)**	**8,856 (7,228–10,484)**	**195 (160–230)**
**Sex**								
Male	97 (76–118)	326 (249–404)	370 (296–444)	469 (372–567)	1,538 (1,258–1,818)	338 (277–398)	5,947 (4,835–7,060)	229 (187–270)
Female	67 (53–81)	234 (181–288)	230 (185–275)	330 (262–397)	871 (714–1,029)	226 (186–267)	2,908 (2,366–3,451)	150 (123–178)
**Type of injury^§^**								
Laceration/Puncture	47 (36–59)	81 (61–102)	146 (115–178)	99 (78–119)	555 (445–665)	66 (53–79)	1,608 (1,290–1,927)	35 (29–42)
Strain/Sprain	28 (22–33)	47 (38–57)	112 (84–140)	75 (57–94)	483 (372–594)	57 (44–70)	2,119 (1,588–2,650)	47 (35–58)
Contusion/Abrasion/Crushing	20 (15–24)	34 (26–42)	86 (66–106)	58 (44–71)	362 (289–435)	43 (35–52)	1,245 (986–1,505)	27 (22–33)
Dislocation/Fracture	11 (7–14)	18 (12–24)	32 (24–39)	21 (16–26)	120 (95–145)	14 (11–17)	526 (440–613)	12 (10–13)
Other/Not stated	59 (44–74)	101 (75–127)	224 (178–271)	151 (120–182)	889 (716–1,061)	106 (86–126)	3,357 (2,678–4,035)	74 (59–89)
**Event or exposure^¶^**								
Contact with objects/equipment	73 (56–91)	125 (95–156)	270 (214–326)	182 (144–219)	985 (796–1,174)	117 (95–139)	2,888 (2,361–3,415)	64 (52–75)
Overexertion/Bodily reaction	27 (21–33)	46 (35–56)	137 (103–171)	92 (69–115)	595 (469–720)	71 (56–85)	2,618 (2,034–3,202)	58 (45–70)
Exposure to harmful substance/ environment	24 (17–31)	41 (29–53)	73 (56–89)	49 (38–60)	294 (234–354)	35 (28–42)	985 (763–1,207)	22 (17–27)
Fall/Slip/Trip	22 (17–28)	38 (29–48)	67 (50–83)	45 (34–56)	260 (207–312)	31 (25–37)	1,126 (915–1,336)	25 (20–29)
Violence/Other injuries by persons or animals	12 (9–15)	20 (15–25)	35 (27–44)	24 (18–30)	194 (150–237)	23 (18–28)	840 (626–1,054)	19 (14–23)
Other events	6 (3–9)	10 (6–15)	19 (14–24)	13 (9–16)	82 (69–95)	10 (8–11)	399 (333–465)	9 (7–10)

**Abbreviations**: CI = confidence interval; FTE = full-time equivalent; NE = national estimate.

* Nonfatal injury rates are per 10,000 FTE workers; one FTE = 2,000 hours worked/year. U.S. Census Bureau's Current Population Survey labor force denominator estimates were used to calculate rates.

^†^ Analysis limited to workers aged 25–44 years to allow a rate comparison with workers who more closely resemble young workers in terms of physical health status.

^§^ Type of injury is defined by the nature of the most severe injury as described by attending physician or other medical staff.

^¶^ Event or exposure is defined by the Bureau of Labor Statistics as the way in which the injury was produced or inflicted.

**FIGURE:**
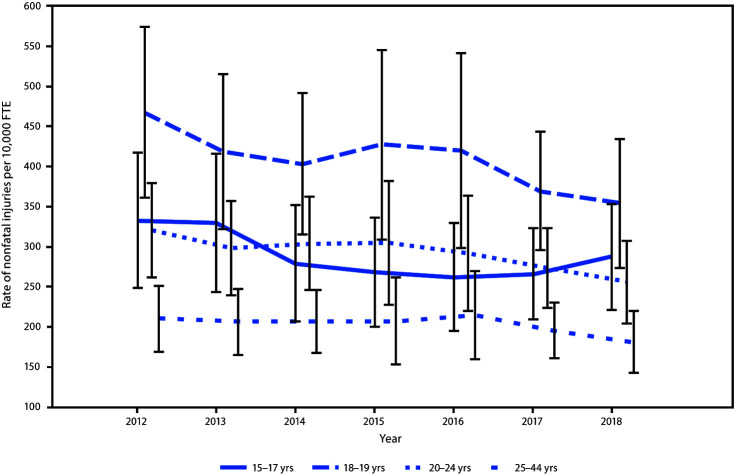
Rate of hospital emergency department–treated nonfatal occupational injuries,* by age group — National Electronic Injury Surveillance System occupational supplement, United States, 2012–2018^†^ **Abbreviation:** FTE = full time equivalent. *Nonfatal injury rates are per 10,000 FTE workers; one FTE = 2,000 hours worked/year. U.S. Census Bureau's Current Population Survey labor force denominator estimates were used to calculate rates. ^†^ With 95% confidence intervals indicated by error bars.

Contact with objects and equipment was the leading cause of occupational ED-treated injuries among all age groups examined, with rates of injuries ranging from 64 per 10,000 FTE among workers aged 25–44 years to 182 per 10,000 FTE among workers aged 18–19 years (Table 1). Lacerations and punctures were the most common type of ED-treated injuries reported among workers aged <25 years, with injury rates ranging from 66 to 99 per 10,000 FTE, whereas strains and sprains were most common among workers aged 25–44 years (injury rate of 47 per 10,000 FTE).

Analyses of SOII data indicate that in 2018, contact with objects or equipment was the leading cause of injury requiring at least 1 day away from work among workers aged 15–17 years (49%), 18–19 years (44%), and 20–24 years (34%), and the leading cause of such injuries among workers aged 25–44 years was overexertion (32%) (Table 2). Among workers aged 15–17 years, those in the leisure and hospitality industry had the highest percentage of work-related injuries requiring at least 1 day away from work (56% of injuries within this age group), with most of these injuries occurring among workers in the accommodation and food services subsector (48% of injuries within this age group). Among workers in age groups 18–19, 20–24, and 25–44 years, those in the trade, transportation, and utilities industry had the highest percentages of injuries requiring at least 1 day away from work, with the largest portions of these injuries occurring among workers in the retail trade subsector. Across all age groups, workers in service occupations had the highest percentages of injuries requiring at least 1 day away from work, including 66% among workers aged 15–17 years.

**TABLE 2:** National estimates*and percentages^†^ of total injuries requiring ≥1 day away from work,^§^ by age group and selected characteristics— Survey of Occupational Injuries and Illnesses,^¶^ United States, 2018**

Characteristic	Age group of worker, yrs							
	15–17		18–19		20–24		25–44^††^	
	NE (95% CI)	%	NE (95% CI)	%	NE (95% CI)	%	NE (95% CI)	%
**Total**	**5,830 (5,510**–**6,150)**	**100**	**21,630 (20,952**–**22,308)**	**100**	**97,050 (95,148–98,952)**	**100**	**461,770 (454,529–469,011)**	**100**
**Sex**								
Male	3,020 (2,795–3,245)	52	13,640 (13,132**–**14,148)	63	60,620 (59,313–61,927)	63	287,480 (282,409–292,551)	62
Female	2,800 (2,586–3,014)	48	7,990 (7,614–8,366)	37	36,250 (35,326–37,174)	37	172,350 (169,310–175,390)	37
**Industry**								
Leisure and hospitality	3,270 (2,956–3,584)	56	4,310 (3,938–4,682)	20	13,520 (12,672–14,368)	14	36,700 (34,758–38,642)	8
Accommodation and food services	2,780 (2,469–3,091)	48	3,600 (3,240–3,960)	17	11,480 (10,670–12,290)	12	30,900 (29,083–32,717)	7
Trade, transportation and utilities	1,000 (878–1,122)	17	7,450 (7,070–7,830)	34	29,770 (28,778–30,762)	31	111,830 (108,761–114,899)	24
Retail trade	940 (817–1,063)	16	4,870 (4,545–5,195)	23	16,420 (15,615–17,225)	17	46,600 (44,591–48,609)	10
Educational and health services	290 (238–342)	5	2,150 (2,003–2,297)	10	13,210 (12,770–13,650)	14	68,160 (66,557–69,763)	15
Health care and social assistance	220 (175–265)	4	1,840 (1,703–1,977)	9	12,230 (11,799–12,661)	13	63,320 (61,707–64,933)	14
Manufacturing	70 (44–96)	1	2,090 (1,938–2,242)	10	9,220 (8,841–9,599)	10	48,640 (47,305–49,975)	11
Construction		0	1,740 (1,467–2,013)	8	7,700 (7,006–8,394)	8	37,990 (35,458–40,522)	8
Professional and business services	350 (255–445)	6	650 (516–784)	3	7,250 (6,596–7,904)	8	27,220 (25,193–29,247)	6
Other services except public administration	320 (185–455)	5	1,280 (966–1,594)	6	2,730 (2,190–3,270)	3	9,680 (8,124–11,236)	2
**Occupation**								
Service	3,870 (3,620–4,120)	66	8,180 (7,795–8,565)	38	30,570 (29,731–31,409)	31	141,840 (139,338–144,342)	31
Transportation and material moving	500 (411–589)	9	4,050 (3,788–4,312)	19	15,840 (15,281–16,399)	16	78,970 (77,267–80,673)	17
Sales and related	460 (374–546)	8	2,090 (1,906–2,274)	10	7,430 (7,066–7,794)	8	20,750 (20,099–21,401)	4
Office and administrative support	210 (153–267)	4	1,210 (1,070–1,350)	6	7,140 (6,790–7,490)	7	27,840 (27,076–28,604)	6
Production	20 (1–39)	0	2,040 (1,860–2,220)	9	7,970 (7,595–8,345)	8	41,690 (40,628–42,752)	9
Construction and extraction	20 (4–36)	0	1,820 (1,649–1,991)	8	8,110 (7,729–8,491)	8	38,900 (37,909–39,891)	8
Installation, maintenance, and repair	100 (61–139)	2	810 (697–923)	4	7,570 (7,199–7,941)	8	36,350 (35,424–37,276)	8
Healthcare practitioners and technical	60 (28–92)	1	240 (178–302)	1	4,230 (3,965–4,495)	4	27,710 (26,950–28,470)	6
**Nature of injury^§§^**								
Cut/Laceration/Puncture	1,630 (1,467–1793)	28	4,680 (4,396–4,964)	22	15,200 (14,664–15,736)	16	45,560 (44,488–46,632)	10
Sprain/Strain/Tear	1,000 (873–1,127)	17	5,170 (4,876–5,464)	24	29,120 (28,321–29,919)	30	162,710 (159,840–165,580)	35
Soreness/Pain	650 (548–752)	11	2,960 (2,740–3,180)	14	15,670 (15,117–16,223)	16	85,800 (84,118–87,482)	19
Bruise/Contusion	330 (258–402)	6	2,400 (2,202–2,598)	11	9,770 (9,349–10,191)	10	40,180 (39,156–41,204)	9
Fracture	580 (483–677)	10	1,270 (1,128–1,412)	6	5,860 (5,538–6,182)	6	32,310 (31,423–33,197)	7
Heat (thermal) burns	620 (520–720)	11	1,050 (920–1,180)	5	2,930 (2,712–3,148)	3	6,670 (6,330–7,010)	1
**Event/Exposure^¶¶^**								
Contact with object/equipment	2870 (2,651–3,089)	49	9,440 (9,033–9,847)	44	33,370 (32,520–34,220)	34	117,960 (115,648–120,272)	26
Overexertion/Bodily reaction	620 (520–720)	11	4,280 (4,012–4,548)	20	23,420 (22,731–24,109)	24	147,350 (144,751–149,949)	32
Fall/Slip/Trip	1,280 (1,137–1,423)	22	4,000 (3,741–4,259)	18	19,030 (18,396–19,664)	20	97,630 (95,716–99,544)	21
Violence/Other injuries by persons or animals	110 (68–152)	2	1,650 (1,488–1,812)	8	8,110 (7,729–8,491)	8	43,100 (42,086–44,114)	9
Exposure to harmful substances/environments***	770 (660–880)	13	1,630 (1,467–1,793)	8	6,050 (5,730–6,370)	6	22,550 (21,843–23,257)	5
Transportation incidents	180 (126–234)	3	590 (493–687)	3	6,510 (6,178–6,842)	7	29,850 (29,031–30,669)	6
**No. of days away from work**								
1	1,390 (1,240–1,540)	24	4,500 (4,227–4,773)	21	17,190 (16,617–17,763)	18	67,140 (65,692–68,588)	15
2	800 (687–913)	14	2,940 (2,721–3,159)	14	13,570 (13,065–14,075)	14	53,980 (52,710–55,250)	12
3–5	1,100 (968–1,232)	19	5,170 (4,876–5,464)	24	19,360 (18,753–19,967)	20	87,350 (85,638–89,062)	19
6–10	650 (548–752)	11	3,050 (2829–3,271)	14	14,020 (13,498–14,542)	14	54,140 (52,867–55,413)	12
11–20	1,150 (1,015–1,285)	20	2,490 (2,290–2,690)	12	10,860 (10,413–11,307)	11	50,620 (49,429–51,811)	11
21–30	130 (85–175)	2	890 (771–1,009)	4	5,120 (4,829–5,411)	5	27,920 (27,154–28,686)	6
≥31	600 (502–698)	10	2,590 (2,387–2,793)	12	16,920 (16,356–17,484)	17	120,620 (118,256–122,984)	26

**Abbreviations:** FTE = full-time equivalent; NE = national estimate.

* Per 10,000 FTE workers; one FTE = 2,000 hours worked/year.

^†^ Only categories and subcategories with ≥5% of all cases for at least one of the age groups are represented in the table; therefore, totals may not sum to 100.

^§^ Includes cases with injuries that result in days away from work with or without restricted work activity.

^¶^ Unpublished data from Survey of Occupational Injuries and Illnesses (SOII), U.S. Department of Labor, Bureau of Labor Statistics (BLS).

** Only the most current year of data (2018) available at the time the analysis was conducted is included because rates and aggregate counts for injuries requiring at least 1 day away from work cannot be calculated for the age groups analyzed for the SOII data.

^††^ Analysis limited to workers aged 25–44 years to allow a rate comparison with workers who more closely resemble young workers in terms of physical health status.

^§§^ Nature of injury is defined by BLS as the physical characteristics of the disabling injury.

^¶¶^ Event or exposure is defined by BLS as the way in which the injury was produced or inflicted.

*** Exposure to harmful substances or environments includes exposure to hot objects or heat burns.

## Discussion

Despite a decline in overall ED-treated injury rates from 2012 to 2018, workers aged 15–24 years experienced higher rates of injury than did workers aged 25–44 years. Consistent with previous analyses (*3*), the highest rate of ED-treated injury occurred among workers aged 18–19 years.

Despite progress toward reducing injury rates among workers aged 15–24 years,^§§§§§^ workers in this age group continue to experience a disproportionately high rate of occupational injury when compared with adults (aged 25–44 years). As reported previously (*3*), within all age groups, higher rates of ED-treated injuries occurred among males than among females. Given that approximately one half of workers aged 15–17 years with a reported injury were employed in the leisure and hospitality industry and that most of these injuries occurred in accommodation and food services, preventive interventions targeting employers in this industry and subsector could reduce work-related injuries among young workers.

The disparity in the number of injuries among young workers has been reported in other countries (*6*,*7*). Evidence suggests that contributors to increased injury risk among younger workers include the following: workplace hazards associated with young worker jobs; violations of child labor laws; fast pace of work; minority status; and lack of skills, experience, supervision, and high-quality safety training. Young workers might be less likely to recognize workplace hazards, voice safety concerns, and be aware of their legal protections (*3*,*6*–*8*).

The findings in this report are subject to at least three limitations. First, NEISS-Work data include only workers treated in EDs and not in other health care settings (*3*), and unpublished SOII data used for analysis capture only those injuries serious enough to require at least 1 day away from work. Thus, both national data sources represent an undercount of the actual prevalence of work-related nonfatal injuries. Second, the two data sources differ substantially in their estimates and methodologies (*1*), and therefore might be considered complementary, but not comparable. Finally, the inability to calculate rates for injuries requiring at least 1 day away from work for the customized age groups analyzed limits characterization of the true magnitude of the work-related injury problem.

A comprehensive, public health strategy is needed for protecting young workers. Employers are responsible for maintaining safe and healthy workplaces, which includes complying with safety, health, and child labor laws; closely supervising young workers; and delivering job-specific safety training. Schools can be a primary venue for providing foundational workplace safety education to youths. NIOSH and its partners developed and evaluated a free curriculum, Talking Safety (*5*,*9*), to teach adolescents workplace safety and health competencies, including identification of workplace hazards and methods for addressing them, how to understand their rights and responsibilities as workers, and how to voice concerns about worker safety issues. Talking Safety has been demonstrated to be effective at educating adolescents on foundational workplace safety competencies, and research provides support for using this curriculum to prepare the future workforce for safe and healthy employment (*9*,*10*). State and federal agencies that perform critical enforcement activities can also promote workplace safety as an essential element of job preparation initiatives. Parents and health care providers can discuss workplace safety topics with their children and patients. Local, state, and federal injury and illness surveillance systems must also provide more comprehensive reporting of the magnitude of injuries to young workers (*2*) to inform development and implementation of evidence-based prevention strategies.

**Summary**

**What is already known about this topic?**

Young workers (aged 15–24 years) experience higher rates of job-related injury than do adult workers (aged 25–44 years).

**What is added by this report?**

During 2012–2018, an estimated 3.2 million nonfatal injuries to young workers were treated in hospital emergency departments, with the highest rates among workers aged 18–19 years. Data from 2018 indicate that the leisure and hospitality industry contributed the highest percentage of injuries to workers aged 15–17 years requiring at least 1 day away from work.

**What are the implications for public health practice?**

A comprehensive, public health strategy for protecting young workers requires designing and maintaining safer worksites, legislation and enforcement, and education and training.

## Footnotes

* https://www.cpsc.gov/Research--Statistics/NEISS-Injury-Data.

† https://www.bls.gov/iif/soii-overview.htm.

§ Per 10,000 full-time equivalent (FTE) workers; one FTE = 2,000 hours worked per year.

¶ Service occupations include those in Standard Occupational Classification groups 31–39, such as health care support occupations, protective service occupations, food preparation and serving related occupations, building and grounds cleaning and maintenance, and personal care and service occupations. https://www.bls.gov/soc/2018/soc_2018_manual.pdf.

** The Consumer Product Safety Commission (CPSC) collects the NEISS-Work data as a supplement to its NEISS surveillance of injuries related to consumer products. The NEISS-Work data are mutually exclusive of the consumer product-related data CPSC collects. Because of hospital closures and nonparticipation, the number of hospitals varied throughout the study period. The present analysis was conducted using raw data files provided to CDC/NIOSH. NEISS-Work data are available from the Work-Related Injury Statistics Query System. https://wwwn.cdc.gov/wisards/workrisqs.

†† Analysis of custom query data with modified age cohorts from SOII. https://www.bls.gov/iif/soii-data.htm#dafw.

§§ Illnesses are excluded in the analysis because they are not captured in NEISS-Work and account for <5% of SOII cases.

¶¶ Cases are included in NEISS-Work when ED personnel identify a work-related injury occurring to a noninstitutionalized, civilian employee working for compensation, working on a farm, or volunteering for an organized group.

*** https://www.osha.gov/recordkeeping/index.html.

††† https://www.census.gov/eos/www/naics.

§§§ https://www.bls.gov/soc/.

¶¶¶ SOII excludes all work-related fatalities and nonfatal work injuries and illnesses for those self-employed, workers on farms with ≤10 employees, private household workers, volunteers, and federal government workers.

****
https://www.bls.gov/iif/oshoiics.htm .

†††† The Fair Labor Standards Act of 1938 child labor provisions prohibit employment of minors in certain jobs and under conditions harmful to their health or well-being. They include restrictions on hours of work for youths aged <16 years and delineate hazardous occupations (so-called “hazardous orders”) for farm (<16 years) and nonfarm jobs (<18 years). Most states have enacted additional protections for working youths. https://www.dol.gov/agencies/whd/compliance-assistance/handy-reference-guide-flsa#9.

§§§§ Limiting the analysis to workers aged 25–44 years allows a rate comparison with workers who more closely resemble young persons in terms of physical health status.

¶¶¶¶ https://www.census.gov/programs-surveys/cps.html.

***** https://wwwn.cdc.gov/wisards/workrisqs/rate.aspx.

††††† The Current Population Survey is collected by the U.S. Census Bureau for the Bureau of Labor Statistics. https://www.bls.gov/iif/osh_rse.htm.

§§§§§ Healthy People 2020 occupational safety and health objective OSH-2.3: reduce work-related injuries among adolescent workers (aged 15–19 years). https://www.healthypeople.gov/2020/topics-objectives/topic/occupational-safety-and-health/objectives.
